# 123 National Study of the Increased Incidence of Complex Regional Pain Syndrome Following Severe Burn Injury

**DOI:** 10.1093/jbcr/irae036.122

**Published:** 2024-04-17

**Authors:** Sunskruthi Krishna, Suhaib Shah, Carolina Segura, Yash Ramgopal, Jean pierre Durand, Christopher G Richter, Steven E Wolf, Amina E I ayadi, Juquan Song

**Affiliations:** University of Texas Medical Branch, Galveston, TX; University of Texas Medical Branch, Blocker Burn Unit, Galveston, TX; University of Texas Medical Branch, Galveston, TX; University of Texas Medical Branch, Blocker Burn Unit, Galveston, TX; University of Texas Medical Branch, Galveston, TX; University of Texas Medical Branch, Blocker Burn Unit, Galveston, TX; University of Texas Medical Branch, Galveston, TX; University of Texas Medical Branch, Blocker Burn Unit, Galveston, TX; University of Texas Medical Branch, Galveston, TX; University of Texas Medical Branch, Blocker Burn Unit, Galveston, TX; University of Texas Medical Branch, Galveston, TX; University of Texas Medical Branch, Blocker Burn Unit, Galveston, TX; University of Texas Medical Branch, Galveston, TX; University of Texas Medical Branch, Blocker Burn Unit, Galveston, TX; University of Texas Medical Branch, Galveston, TX; University of Texas Medical Branch, Blocker Burn Unit, Galveston, TX; University of Texas Medical Branch, Galveston, TX; University of Texas Medical Branch, Blocker Burn Unit, Galveston, TX

## Abstract

**Introduction:**

Complex Regional Pain Syndrome (CRPS) is a clinical condition that presents after an operation, trauma, or burn injury with allodynia, hyperalgesia, and myasthenia. The etiology of CRPS remains poorly understood and there is a notable gap in literature linking the severity of burns to the risk of developing CRPS. Our study aims to address this by investigating retrospective patient data to determine the relationship between the degree and total body surface area (TBSA) of burn injury and the incidence of CRPS.

**Methods:**

Patient records were accessed through a national database of de-identified electronic patient medical records from 2010 to 2019. Specific ICD-10 codes were used to define cohorts and outcomes. Three cohorts were established among patients with a history of burns- those with first-, second-, and third-degree burns. Two additional cohorts of TBSA of burns of ≤20% and >20% were established. These cohorts were analyzed for the risk of developing CRPS (type 1 and 2) within 6 months of first instance of burn injury. A z-test was performed after propensity score matching with age, sex, race, and ethnicity with significant p-value < 0.05.

**Results:**

0.274% of third-degree burn patients (n= 2,682), 0.213% of second-degree burn patients (n= 2,009), and 0.221% of first-degree burn patients (n= 2,686) were diagnosed with CRPS within 6 months of the first instance of burn injury. Patients with third-degree burns were at a significantly higher risk for developing CRPS compared to patients with first-degree burns (p= 0.0270), second-degree burns (p=0.0102), and first- and second-degree burns combined (p < 0.001) within 6 months post-burn injury. Patients with more severe burns of TBSA>20% (n= 6,628) were more likely to develop CRPS with a risk of 0. 422% (p= 0.0305). Additionally, burn patients with CRPS were more likely to be female than without CRPS for first- (74%), second- (67%), and third- degree burns (53%), and TBSA>20% (62%).

**Conclusions:**

Patients who undergo third-degree burns or burns of TBSA>20% have a significantly greater risk for developing CRPS post-injury. Based on hypothesized mechanisms, the extensive tissue damage of more severe burns will result in a higher incidence of CRPS.

**Applicability of Research to Practice:**

These results indicate a need for a proactive risk assessment and more comprehensive post-hospital care to limit and manage the risk of CRPS in burn patients. Future research can inquire about other predisposing factors and complications of CRPS in the burn population and explore incidence of CRPS for other time periods post-injury.

